# Dissociation between AKAP3 and PKA_RII_ Promotes AKAP3 Degradation in Sperm Capacitation

**DOI:** 10.1371/journal.pone.0068873

**Published:** 2013-07-23

**Authors:** Pnina Hillman, Debby Ickowicz, Ruth Vizel, Haim Breitbart

**Affiliations:** The Mina & Everard Goodman Faculty of Life Sciences, Bar-Ilan University, Ramat-Gan, Israel; Institute of Zoology, Chinese Academy of Sciences, China

## Abstract

Ejaculated spermatozoa must undergo a series of biochemical modifications called capacitation, prior to fertilization. Protein-kinase A (PKA) mediates sperm capacitation, although its regulation is not fully understood. Sperm contain several A-kinase anchoring proteins (AKAPs), which are scaffold proteins that anchor PKA. In this study, we show that AKAP3 is degraded in bovine sperm incubated under capacitation conditions. The degradation rate is variable in sperm from different bulls and is correlated with the capacitation ability. The degradation of AKAP3 was significantly inhibited by MG-132, a proteasome inhibitor, indicating that AKAP3 degradation occurs via the proteasomal machinery. Treatment with Ca^2+^-ionophore induced further degradation of AKAP3; however, this effect was found to be enhanced in the absence of Ca^2+^ in the medium or when intracellular Ca^2+^ was chelated the degradation rate of AKAP3 was significantly enhanced when intracellular space was alkalized using NH_4_Cl, or when sperm were treated with Ht31, a peptide that contains the PKA-binding domain of AKAPs. Moreover, inhibition of PKA activity by H89, or its activation using 8Br-cAMP, increased AKAP3 degradation rate. This apparent contradiction could be explained by assuming that binding of PKA to AKAP3 protects AKAP3 from degradation. We conclude that AKAP3 degradation is regulated by intracellular alkalization and PKA_RII_ anchoring during sperm capacitation.

## Introduction

Ejaculated sperm are incapable of fertilizing an oocyte before undergoing a series of biochemical and physiological changes in the female reproductive tract, collectively known as capacitation [1]. Capacitation confers upon the sperm the ability to gain hyperactive motility, and interact with the egg to undergo the acrosome reaction (AR) a process that enables the sperm to penetrate and fertilize the oocyte [1].

Several molecules are required for successful capacitation and in vitro fertilization; these include bicarbonate, serum albumin and Ca^2+^. Bicarbonate activates the sperm protein soluble adenyl cyclase (SACY), which results in increased levels of cAMP and cAMP-dependent protein kinase (PKA) activity [2–4]. PKA is a tetrameric enzyme consisting of two catalytic subunits (C), which are maintained in an inactive state by binding to a regulatory (R) subunit homodimer. Thus, two PKA subtypes exist depending on the RI or RII regulatory subunits forming the regulatory homodimeric component. Upon binding of cAMP to the regulatory subunits of type I- or type II-holoenzyme, the catalytic subunits are released as active serine–threonine kinases and can phosphorylate their specific substrates, initiating a cascade of signaling events inside the cell [5–8]. PKA was shown to be involved in sperm capacitation; in bovine sperm, when PKA is blocked, no capacitation occurs [9]. PKA mediates the activation of PI3K [10,11] and PLD [9] in bovine sperm capacitation. The activation of PKA modulates the response of calcium channels such as CatSper [12]. It has been shown that PKA inhibition blocks the onset of tyrosine phosphorylation of many proteins [3], and PKA is involved in regulation of sperm motility [13,14].

A-kinase anchoring proteins (AKAPs) contribute to the specificity as well as the versatility of the cAMP-PKA pathway by assembling multiprotein signal complexes, allowing signal termination by phosphatases and cross-talk between different signaling pathways in close proximity to the substrates [15,16]. AKAPs belong to a family of scaffolding proteins, which provide a key mechanism enabling a common signaling pathway to serve many different functions. Sequestering a signaling enzyme to a specific sub-cellular environment not only ensures that the enzyme is near its relevant targets, but also segregates this activity to prevent indiscriminate phosphorylation of other substrates. These anchoring proteins form multi-protein complexes that integrate cAMP signaling with other pathways and signaling events [17].

Most AKAPs contain a recognition sequence that forms a binding site for the regulatory subunits of PKA. To date, over 50 AKAPs have been identified in mammals and lower organisms [18]. One of the first physiological roles identified for AKAPs was in learning and memory; AKAP79 (or its mouse ortholog, AKAP150) can regulate synaptic plasticity through regulation of the neuron channel response. This is accomplished by AKAP79 through scaffolding, targeting, and regulation of the signaling molecules PKA, protein kinase C (PKC) and protein phosphatase 2B (PP2B, calcineurin) at the postsynaptic membrane [19].

In addition to scaffolding PKA, AKAPs also bind to a group of four proteins that share homology to the RII dimerization/docking (R2D2) domain. Proteins with the R2D2 domain are expressed at high levels in the testis. These proteins function in the regulation of flagella and cilia independent of PKA activity and, unlike RII, do not bind cAMP [20].

AKAPs play important roles in sperm function, including regulation of motility, sperm capacitation, and the acrosome reaction. Several AKAPs have been identified in sperm, including AKAP84 (AKAP1), AKAP110 (AKAP3), AKAP82 (AKAP4), AKAP95 (AKAP8), AKAP220 (AKAP11), gravin (AKAP250; AKAP12), WAVE-1, and MAP2 [4,21–24].

The AKAP3 and AKAP4 isoforms are uniquely expressed by spermatids and spermatozoa, localize in the flagellum, and are involved in sperm motility. AKAP3 is mainly localized at the principal piece of the tail [25]. AKAP4 knockout mice are immotile and infertile. AKAP4 is sperm-specific, and is the major fibrous sheath protein of the principle piece of sperm flagellum [26].

A yeast two-hybrid screen using AKAP3 as bait identified AKAP-associated sperm protein and ropporin as two proteins that interact with the amphipathic helix in a manner similar to PKA. These proteins have been named R2D2 proteins [24]. Sperm from AKAP-associated sperm protein and ropporin double knockout mice are immotile and have obvious defects in sperm morphology, accompanied by a reduction in AKAP3 levels [27].

Several recent studies illustrate regulation of the AKAP/PKA interaction via phosphorylation. Luconi et al. demonstrated that tyrosine phosphorylation of AKAP3 enhances sperm motility by increasing its binding to PKA [28]. Although the intracellular regulation of PKA activity during sperm capacitation has been extensively described, specific biological functions for AKAPs have been harder to identify. Therefore, our aim in the present study was to identify the behavior of AKAP3 during sperm capacitation.

## Materials and Methods

### Materials and Antibodies

MG-132, protease inhibitor cocktail III, ProteoExtract Sub-cellular Proteome Extraction Kit, BAPTA-AM, A23187, and BLOT-QuickBlocker reagent were purchased from Calbiochem (San Diego, CA, USA). St-HT31 and St-Ht31-P peptides were purchased from Promega (Madison, WI, USA). Rabbit polyclonal antibody against AKAP3 was kindly gifted by Dr. Daniel Carr (Portland, OR, USA). Rabbit polyclonal anti-AKAP95 (R-146) and mouse monoclonal β-actin (C4) HRP were purchased from Santa Cruz biotechnology (California, USA). PKA, RII (C-term) rabbit antibody was purchased from Epitomics (Burlingame, California). Goat anti mouse IgG-HRP conjugated and goat anti rabbit IgG-HRP conjugated were purchased from Bio-Rad (Richmond, CA, USA). Re-Blot plus Strong Solution (×10) was purchased from Millipore (Temecula, California, 92590). All other chemicals were purchased from Sigma (Sigma-Aldrich Israel Ltd, Rehovot, Israel) unless otherwise stated.

### Sperm Preparation

Ejaculated bull spermatozoa were obtained by using artificial vagina, and the ‘swim up’ technique was applied to obtain motile sperm. Bovine sperm was supplied by the SION Artificial Insemination Centre (Hafetz-Haim, Israel). Sperm cells were washed three times by centrifugation (780g for 10 min at 25°C) in NKM buffer (110 mM NaCl, 5 mM KCl, and 20 mM 3-N-morpholino propanesulfonic acid (MOPS) (pH 7.4)) and the sperm were allowed to swim up after the last wash. The washed cells were counted and maintained at room temperature until use. Only sperm preparations that contained at least 80% motile sperm were used in the experiments; motility was not significantly reduced at the end of the incubations.

### Sperm Capacitation

*In vitro* capacitation of bovine sperm was induced as described previously [29]. Briefly, sperm pellets were resuspended to a final concentration of 10^8^ cells/ml in mTALP (Modified Tyrode solution) medium (100 mM NaCl, 3.1 mM KCl, 1.5 mM MgCl_2_, 0.92 mM KH_2_PO_4_, 25 mM NaHCO_3_, 20 mM Hepes (pH 7.4), 0.1 mM sodium pyruvate, 21.6 mM sodium lactate, 10 IU/ml penicillin, 1 mg/ml BSA, 20 µg/ml heparin, and 2 mM CaCl_2_). The cells were incubated in this capacitation medium for 4h at 39°C in 0.5% CO_2_ atmosphere. The capacitation state of the sperm was routinely confirmed after the 4h incubation in mTALP by examining the ability of the sperm to undergo the acrosome reaction by the addition of A23187 (10µM) or epidermal growth factor (EGF, 1 ng/ml).

### Assessment of sperm acrosome reaction

Washed cells (10^8^ cells/ml) were capacitated for 4 h at 39°C in mTALP medium. Inducers were then added for another 20 min of incubation. The percentage of acrosome-reacted sperm was determined microscopically on air-dried sperm smears using FITC-conjugated Pisum sativum agglutinin (PSA). An aliquot of spermatozoa (10^6^ cells) was smeared on a glass slide and allowed to air-dry. The sperm were then permeabilized by methanol for 15 min at room temperature, washed three times at 5-min intervals with TBS, air dried, and then incubated with FITC-PSA (50 µg/ml in TBS) for 30 min, washed twice with H_2_O at 5-min intervals, and mounted with FluoroGuard Antifade (Bio-Rad Lab). For each experiment, at least 200 cells per slide on duplicate slides were evaluated (total of 400 cells for one experiment). Cells with green staining over the acrosomal cap were considered acrosome intact; those with equatorial green staining or no staining were considered acrosome reacted.

### Immunoblot analysis

Sperm were washed by centrifugation for 5 min at 10,000 ×g at 4°C, and then the supernatant was discarded and the pellet was resuspended in TBS and centrifuged again in order to remove remaining traces of BSA. Sperm lysates were prepared by the addition of lysis buffer (50 mM Tris-HCl pH 7.5, 150 mM NaCl, 6% SDS, protease inhibitor cocktail 1:100, 50 µM NaF, 50 µM pyrophosphate, 0.2 mM Na _3_VO_4_ and freshly added 1 mM phenylmethylsulfonyl fluoride (PMSF)), to the pellet, and the lysate vortexed vigorously for 15 min at room temperature. Lysates were then centrifuged for 5 min at 10,000 ×g at 4°C, the supernatant was transferred, and the protein concentration was determined by the BCA method [30]. Sample buffer ×5 was added to the supernatant, and boiled for 5 min. The extracts were separated on 7% or 10% SDS-polyacrylamide gels and then electrophoretically transferred to nitrocellulose membranes. Blots were routinely stained with Ponceau solution to confirm equal loading and even transfer. The blots were blocked with 1% BSA in Tris-buffered saline, pH 7.6, containing 0.1% Tween 20 (TBST) or 5% BLOT-QuickBlocker in TBST (for the AKAP3 antibody), for 30 min at room temperature. The membranes were incubated overnight at 4°C with the primary antibodies diluted in 1% BSA in TBST. Next, the membranes were washed three times with TBST and incubated for 1h at room temperature with specific horseradish peroxidase (HRP)-linked secondary antibodies (BioRad Lab., Richmond, CA), diluted 1:5000 in TBST and 1% BSA. The membranes were washed three times with TBST and visualized by enhanced chemiluminescence (Amersham, Little Chalfont, UK).

## Results

### AKAP3 is degraded during sperm capacitation

**Figure 1:**
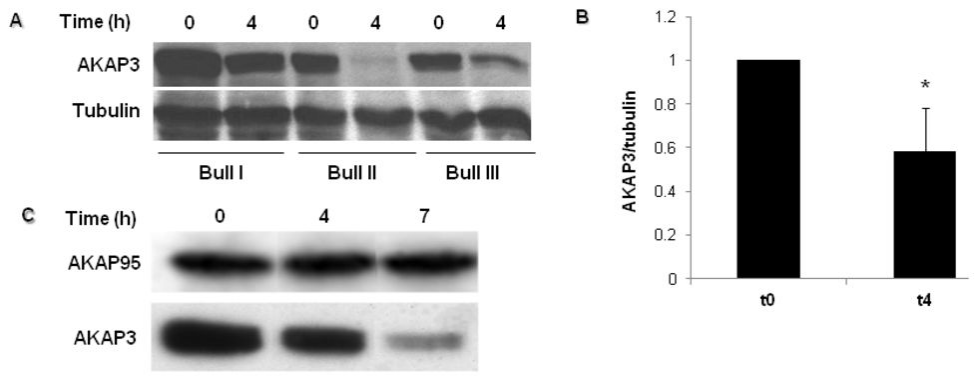
Detection of AKAP3 levels during capacitation. (**A**) Bovine spermatozoa were incubated in capacitation medium. Samples were removed at the beginning and after 4h of capacitation. Proteins were extracted and analyzed by western blot using anti AKAP3 antibody and anti-α-tubulin antibody (loading control). (**B**) Densitometric analysis of AKAP3 bands. The graph represents an average of six different experiments ± SD at each time point. *Significance P< 0.01. (**C**) Bovine spermatozoa were incubated in capacitation medium. Samples were removed at the beginning, and after 4h and 7h of capacitation. Proteins were extracted and analyzed by western blot using anti-AKAP3 and anti-AKAP95 antibodies. The result represents three independent experiments.

Incubation of bovine sperm for 4 hours under capacitation conditions revealed a significant decrease in the levels of AKAP3 (Fig. 1A,B). At the end of the incubation, we could not detect any significant decrease in sperm motility or any signs of increased cell death, demonstrating that the decrease in AKAP3 levels is not due to cell death. The basal levels of sperm AKAP3 differ among bulls as does the rate of degradation of the protein during capacitation. Using densitometry, we found a decrease of 25%, 80% and 40% of AKAP3 protein levels, in bulls I, II, and III respectively. These results are representative of observations made in a larger group of samples (not shown) We found a positive relationships between the decreasing rate of AKAP3 levels during capacitation and the induced acrosomal reaction rate, as a measure for capacitation, in sperm from different bulls. So far we tested sperm from 7 bulls, and the data reveal that we can divide the bulls into two groups: One group (3 bulls) showed >90% decrease in the amount of AKAP3 after 4h incubation under capacitation conditions and >23% acrosome reaction induced after 4h of incubation by epidermal growth factor (EGF). The second group (4 bulls) showed <70% decrease in AKAP3 levels during capacitation and <10% acrosome reaction induced by EGF. No correlation is seen between the decrease in AKAP3 levels during capacitation and the spontaneous acrosome reaction after 4h of incubation. Interestingly, the level of AKAP95 did not change during capacitation (Figure 1C).

**Figure 2:**
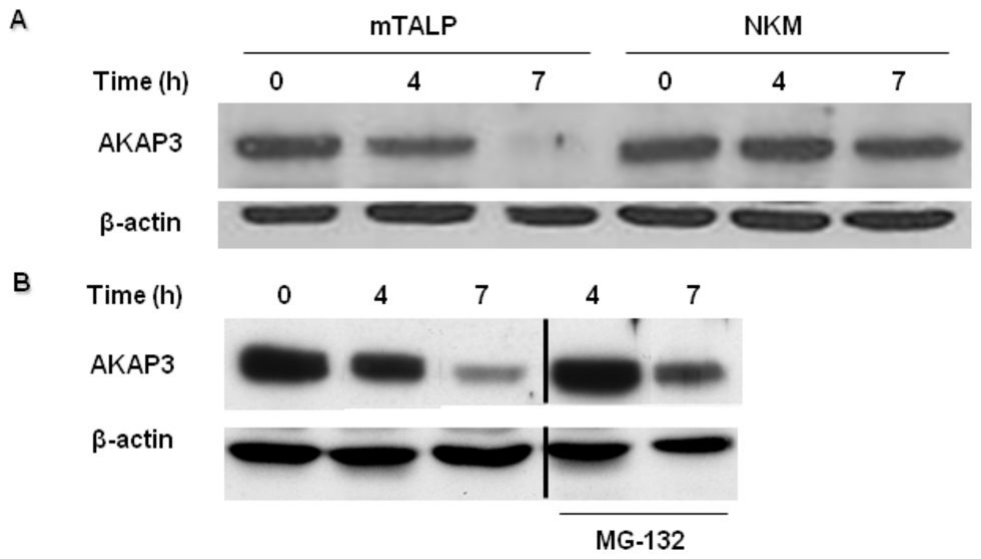
AKAP3 degradation is dependent on capacitation conditions and proteasome activity. Bovine spermatozoa were incubated for 4h or 7h in the mTALP capacitation medium (A and B) or NKM medium (non-capacitating medium) (**A**), in the presence or absence of MG-132 (10 µM) (**B**). Proteins were extracted and analyzed by western blot using anti-AKAP3 and anti-β-actin antibodies. The results represent three independent experiments. Note: The bands from the sides of the cut are from the same blot. We see the cut because few irrelevant bands were taken out of the picture.

To determine whether the decrease of AKAP3 levels depends on capacitation conditions, we incubated the sperm cells in a medium which is not permissive for capacitation. AKAP3 levels remained unchanged during 4-7h of incubation in non-capacitation (NKM) medium, while in capacitation medium (mTALP), AKAP3 levels decreased with incubation time (Figure 2A).

**Figure 3:**
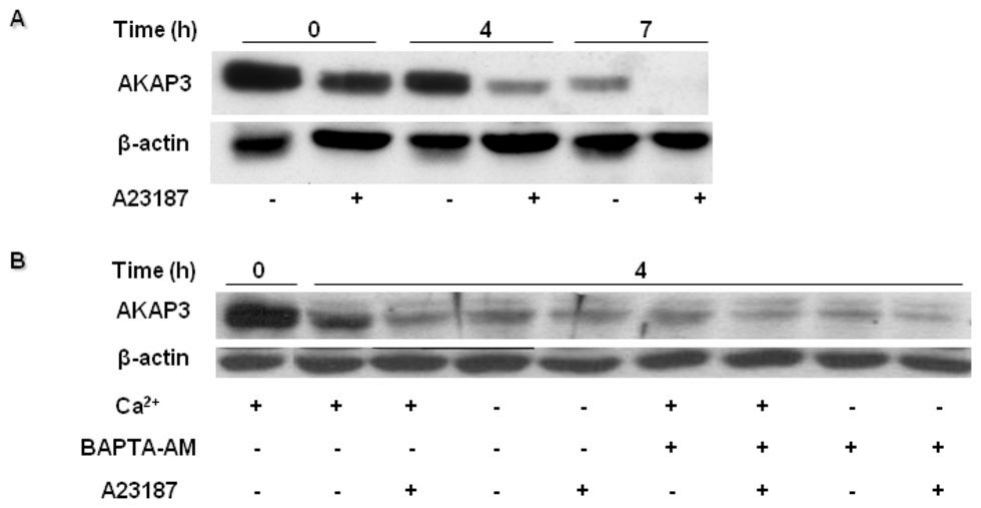
Ca^2+^ ionophore induces AKAP3 degradation. Bovine spermatozoa were incubated in capacitation medium for 4-7h. (**A**) Ca^2+^ ionophore A23187 (10 µM) was added for 20 min at the indicated times. (**B**) The cells were incubated in NKM medium (starvation) for 1h and then transferred to capacitation medium. Spermatozoa were incubated with or without calcium (2 mM) and BAPTA-AM (3 µM) for 4h. Proteins were extracted and analyzed by western blot using anti-AKAP3 antibody and anti-β-actin antibody (loading control). The results represent three independent experiments.

It was shown that proteasomal activity is involved in human sperm capacitation [31,32]. We therefore investigated whether the decrease of AKAP3 levels is mediated by proteosomal activity. Indeed, the decrease of AKAP3 after 4h or 7h of incubation was significantly but not completely inhibited by the proteasome inhibitor, MG-132 (Figure 2B), indicating that AKAP3 degradation occurs via the proteasome machinery. The incomplete inhibition by MG-132 suggests that other mechanism(s) are involved in AKAP3 degradation. It is known that Ca^2+^-dependent proteases such as calpain are present in sperm cells [33]. Therefore, we assumed that the elevation in intracellular calcium concentration during capacitation might play a role in regulating AKAP3 degradation. Elevation of intracellular Ca^2+^ was induced by incubating the sperm cells with the Ca^2+^-ionophore A23187, a well-known Ca^2+^/2H^+^ exchanger. Treatment with the Ca^2+^-ionophore induced further decrease of AKAP3 levels at the beginning as well as at the end of capacitation period (Figure 3A). These data may suggest that AKAP3 degradation is Ca^2+^-dependent.

### Intracellular alkalization, but not calcium influx, regulates AKAP3 degradation

**Figure 4:**
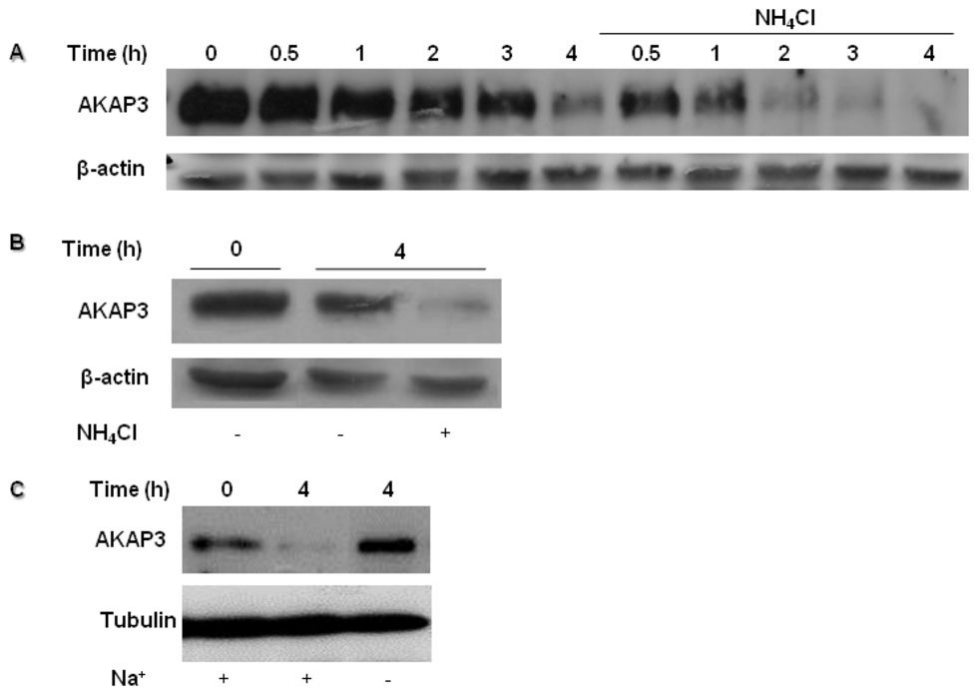
AKAP3 degradation is dependent on alkalization. Bovine spermatozoa were incubated with or without NH_4_Cl (10 mM) for 4h. Proteins were extracted at the indicated times (**A**). Spermatozoa were incubated for 4h NKM medium (non-capacitation medium), in the presence or absence of NH_4_Cl (10 mM) (**B**). Spermatozoa were incubated for 4h in capacitation medium with or without sodium (**C**). Proteins were extracted and analyzed by western blot using anti-AKAP3 antibody and anti-β-actin antibody or anti-tubulin (quantity control). The results represent five independent experiments.

To determine the Ca^2+^-dependence of AKAP3 degradation, we incubated the cells in capacitation medium without adding Ca^2+^, or in the presence of the intracellular Ca^2+^-chelator BAPTA-AM. Surprisingly, we found that A23187 induced further degradation of AKAP3 after 4 h of incubation in Ca^2+^-deficient medium or in BAPTA-AM treated sperm (Figure 4B). Moreover, the reduction of intracellular Ca^2+^ concentrations enhanced degradation of AKAP3. These results do not support the Ca^2+^-dependence of AKAP3 degradation. Alternatively, it is possible that the Ca^2+^ ionophore, which acts as a Ca^2+^/2H^+^ exchanger, can elevate intracellular pH in addition to elevation of [Ca^2+^]_i_. During capacitation, intracellular pH is elevated [34]. To test whether intracellular alkalization would affect AKAP3 degradation, we incubated the sperm cells in capacitation medium in the presence of NH_4_Cl, which elevates intracellular pH. NH_4_Cl significantly enhanced the rate of AKAP3 degradation, which could be detected after a relatively short (30 min) incubation period (Figure 4A). Interestingly, incubation with NH_4_Cl can cause degradation even in medium that does not permit capacitation to occur (Figure 4B). Treatment with NH_4_Cl did not affect sperm motility or spontaneous AR (not shown). One of the mechanisms that mediate intracellular alkalization in sperm cells involves a Na^+^/H^+^ exchanger [35–37]. To determine whether this exchanger is involved in the alkalization that leads to AKAP3 degradation, we incubated the cells in a medium lacking Na^+^ for 4h of capacitation, conditions by which the Na^+^/H^+^ exchanger is inactive. AKAP3 degradation was blocked by incubation in Na^+^ deficient medium (Figure 4C). These results suggest that AKAP3 degradation depends on intracellular alkalization.

### PKA activity does not regulate AKAP3 degradation

**Figure 5:**
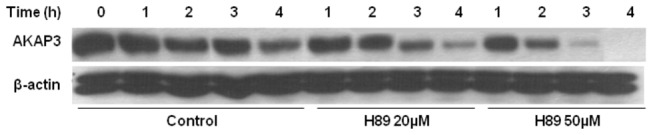
H89 enhances AKAP3 degradation during sperm capacitation. Bovine spermatozoa were incubated for 4h in capacitation medium in the presence or absence of H89 at the indicated concentrations. Proteins were extracted at the indicated times and analyzed by western blot using anti-AKAP3 antibody and anti-β-actin antibody (quantity control). The results represent five independent experiments.

AKAPs are scaffold proteins that anchor PKA, essential for PKA localization in its activation site in the cell. It has been shown elsewhere that PKA activity in bovine sperm increases during capacitation [38]. Moreover, we showed that PKC and PP1γ2 degradation in bovine sperm capacitation depend on PKA activity [38]. Thus, we hypothesized that PKA mediates AKAP3 degradation. To test this possibility, we incubated the sperm cells in the presence of the PKA inhibitor H89, and followed its effect on AKAP3 degradation. To our surprise, we found that H89 did not inhibit, but rather accelerated, AKAP3 degradation in a dose and time dependent manner (Figure 5). In control cells significant degradation of AKAP3 occurred after 4h of incubation, whereas in the presence of 20 µM or 50 µM H89 significant degradation was detected already after 2h or even 1h, respectively (Figure 5). Thus, inhibition of PKA activity induces AKAP3 degradation during capacitation.

**Figure 6:**
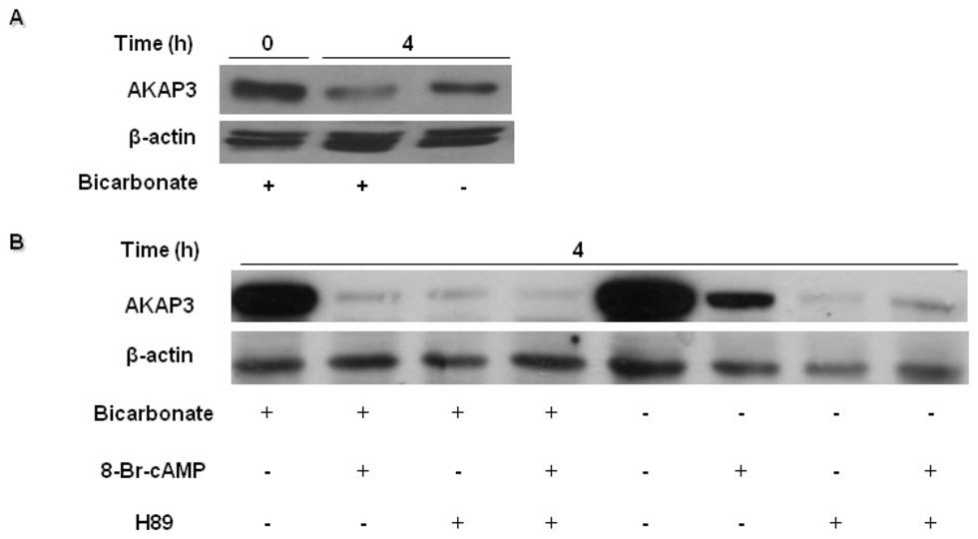
AKAP3 degradation is independent of PKA activation, but depends on PKA anchoring. Bovine spermatozoa were incubated in capacitation medium with or without bicarbonate, H89 (50 µM) or 8Br-cAMP (1 mM) (**A**,**B**). H89 was added at the beginning of capacitation for 10 min, followed by addition of 8Br-cAMP. Samples were removed at the beginning of the assay, and after 4h of capacitation. Proteins were extracted and analyzed by western blot using anti-AKAP3 antibody and anti-β-actin antibodies (loading control). The results represent three independent experiments.

**Figure 7:**
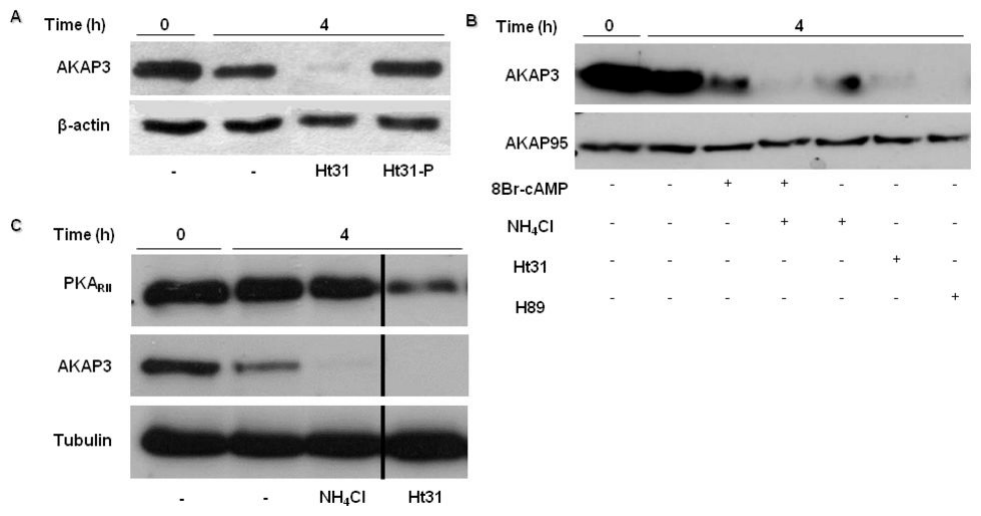
Disruption of PKA anchoring, but not AKAP3 degradation, affects PKA_RII_ state. Bovine spermatozoa were incubated in capacitation medium. Ht31 (5 µM), Ht31-P (5 µM) (control peptide) (**A**,**C**), NH_4_Cl (10 mM) (**B**,**C**), H89 (50µM) and 8Br-cAMP (1 mM) (**B**) were added at the beginning of capacitation. Samples were taken out at the beginning and after 4h of capacitation. Proteins were extracted and analyzed by western blot using anti-AKAP3 (**A**,**B**,**C**), anti-AKAP95 (**B**), anti PKA_RII_ (**C**), and anti-β-actin or anti-α-tubulin antibodies (loading control). The results represent three independent experiments. Note: The bands from the sides of the cut are from the same blot. We see the cut because few irrelevant bands were taken out of the picture.

To further confirm the effect of PKA on AKAP3 levels, cells were incubated in capacitation medium without added bicarbonate, conditions under which sperm PKA is not activated. Most of the sperm cAMP is produced by the soluble-bicarbonate-dependent adenylyl cyclase [39]. Thus, incubating sperm in bicarbonate deficient medium would prevent PKA activation, while activity may be restored by adding the membrane permeable 8Br-cAMP analog. Incubation in bicarbonate-deficient medium partially inhibited AKAP3 degradation (Figure 6A), while a considerable increase in the degradation rate was observed following addition of 8Br-cAMP or the PKA inhibitor H89 (Figure 6B). The enhanced effect of 8Br-cAMP on AKAP3 degradation could suggest that PKA activation mediates this degradation; however, the inhibition of PKA by H89 which also causes a significant acceleration of AKAP3 degradation suggests that AKAP3 degradation is not dependent on PKA activation. Bicarbonate may accelerate AKAP3 degradation due to intracellular alkalization, as suggested above in Figure 4A. These results led us to conclude that besides alkalization, an additional pathway is activated by 8Br-cAMP and by H89, which leads to AKAP3 degradation. Moreover, when cells were incubated in the presence of 8Br-cAMP together with NH_4_Cl, we detected a synergistic enhancement of AKAP3 degradation (Figure 7B).

### Dissociation between PKAR_II_ and AKAP3 promotes AKAP3 degradation

In light of our results that showed no correlation between PKA activity and AKAP3 degradation, we suggested that the binding of PKA to AKAP3 can protect and retard the degradation rate of AKAP3 during capacitation. In order to investigate this possibility, we used an anchoring disrupting peptide (Ht31) which contains the anchoring motif that is present in all AKAPs and competes with AKAPs for binding PKA_RII_. The addition of Ht31 causes immediate inhibition of sperm motility [40].

Cells were incubated in capacitation medium in the presence of the Ht31 peptide for 4h, revealing significantly enhanced AKAP3 degradation during capacitation (Figure 7A). A control peptide that is unable to bind PKA did not affect AKAP3 level (Figure 7A), indicating the specificity of Ht31. In addition, Ht31 did not affect AKAP95, nor did 8Br-cAMP, NH_4_Cl or H89 (Figure 7B). These results support our assumption that PKA anchoring regulates AKAP3 degradation during capacitation.

AKAPs anchor PKA through binding the regulatory subunits of PKA (PKA_RII_). Thus, we wished to characterize the state of PKA_RII_ under the conditions that enable AKAP3 degradation. We found that after sperm treatment with Ht31 for 4h under capacitation conditions, the quantity of PKA_RII_ decreased, but not as drastically as seen for AKAP3 (Figure 7C). When cells were treated with NH_4_Cl, which also enhanced AKAP3 degradation, PKA_RII_ was not affected. These results are due to the fact that Ht31 disrupts all AKAP-PKA interactions, with no specificity to AKAP3; thus, the complete disruption of PKA anchoring affects the state of PKA_RII_, whereas the disruption of AKAP3 alone does not.

## Discussion

AKAPs play important roles in sperm function, including regulation of motility, sperm capacitation, and the acrosome reaction [21,23,24,41]. In the present study we showed that the level of AKAP3 in sperm is decreased during incubation under conditions which induce capacitation (Figure 1A). This decrease in AKAP3 levels during capacitation is due to AKAP3 degradation, a conclusion based on the finding in which the proteasomal inhibitor MG-132 caused significant inhibition of the decrease in AKAP3 levels (Figure 2B). Another possible explanation for the decrease of the amount of AKAP3 after 4h of incubation under capacitation conditions could be its release from the acrosomal region as a result of spontaneous acrosome reaction. This possibility is ruled out due to the fact that the spontaneous acrosome reaction is not correlated with the decrease of AKAP3 levels. Moreover, at time zero, the Ca^2+^-ionophore, A23187, induced AKAP3 degradation but was not able to induce the acrosome reaction. Furthermore, AKAP3 was not present in concentrated media of acrosome reacted cells. Therefore, we suggest that this decrease in AKAP3 protein levels is not due to the release of the protein from the cell as part of acrosomal exocytotic events. These data together with the fact that the proteasome inhibitor, MG-132, caused significant inhibition in AKAP3 degradation during capacitation (Figure 2B), clearly indicate that AKAP3 is degraded during sperm capacitation. It was suggested elsewhere that proteasome activity depends on PKA activity [42]. We showed here that no decrease in AKAP3 is observed when sperm are incubated under non-capacitative conditions (Figure 2A). It is well accepted that sperm PKA in not activated under non-capacitative conditions [35], thus we do not expect to see proteasomal activation, or decrease in AKAP3 amount. This information supports further our conclusion regarding AKAP3 degradation during sperm capacitation. Interestingly, no change was detected in AKAP95 levels (Figure 1C), indicating that AKAP3 degradation is specific and is likely to have physiological importance in the capacitation process.

**Figure 8:**
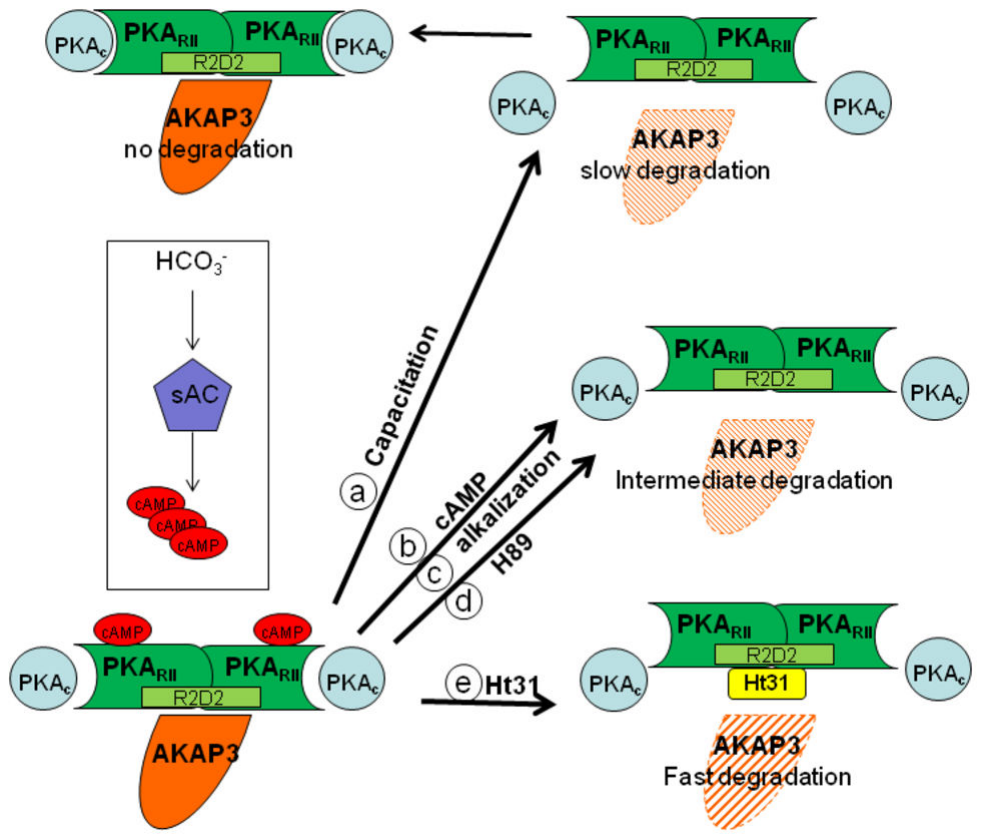
Model: AKAP degradation is dependent on PKA anchoring. At the beginning of capacitation (top left), the AKAP3-PKA_RII_ - PKA_C_ complex is intact. Under these conditions AKAP3 is not degraded. During the capacitation process, bicarbonate activates sAC to generate cAMP which binds to the regulatory subunits (PKA_RII_) (bottom left). This binding releases PKA_C_ from the complex resulting in its activation (top right). When the AKAP3 complex lacks PKA_C_, slow and partial degradation of AKAP3 occurs (a). The release of PKA_C_ is enhanced by cAMP, as is the degradation of AKAP3 (b). For better understanding the data, the various treatments by H89 or intracellular alkalization which accelerate AKAP3 degradation are also included here (c-d, middle right). Disruption of the association between AKAP3 and PKA_RII_ by Ht31, a competing peptide, results in the rapid and complete degradation of AKAP3 (e) (bottom right).

Our data reveal that higher degradation of AKAP3 during capacitation is correlated with higher capacitation ability of the sperm detected by the sperm ability to undergo EGF-induced acrosome reaction. Moreover, it has been shown elsewhere that inhibition of proteasomal activity inhibit sperm from undergoing the capacitation process [32]. These data, although not conclusively prove but support the possible involvement of AKAP3-degradation in sperm capacitation. The finding of a specific inhibitor for AKAP3-degradation would provide a conclusive answer to this question. The mechanisms that regulate AKAP3 degradation are described in our model (Figure 8) which integrates published data together with new data from the present study. During sperm capacitation bicarbonate activates sperm sAC to produce cAMP which activates PKA_C_ by releasing it from the AKAP complex [5–7]. Under capacitation conditions AKAP3 is degraded towards the end of the capacitation (Figures 1, 4, 8a) We have previously shown that PKA activity was increased during incubation of bovine sperm in capacitation medium and under these conditions we find PKA-dependent degradation of PKC and PP1 [38]. In this study we showed that activation of PKA increased AKAP3 degradation (Figures 6B, 8b), however to our surprise, we found that inhibition of PKA activity by H89 does not block, but rather accelerates, AKAP3 degradation (Figures 5, 8d). Moreover, when cAMP/PKA activation is prevented by incubating the cells in bicarbonate deficient medium, AKAP3 degradation is inhibited and degradation could be recover by adding 8Br-cAMP to these cells (Figure 6B). It is well known that cyclic-AMP activates PKA by inducing separation of the two catalytic subunits from the two regulatory subunits [5–7] Under these conditions the AKAP3 free of PKA is highly sensitive to degradation. We suggest that H89 which interacts with the ATP site of the catalytic subunit, accelerates AKAP3 degradation by blocking the re-association of the catalytic subunits with the regulatory subunits causing an increase in PKA-free AKAP3. Indeed, the addition of 8Br-cAMP induced a higher rate of AKAP3 degradation, which was further enhanced by adding H89 (Figure 6B). These data indicate that AKAP3 free of bound PKA is highly sensitive to degradation. This conclusion is further supported by showing that Ht31, an anchoring disrupting peptide also enhances AKAP3 degradation (Figures 7A, 8e). Ht31 also enhances PKA_RII_ degradation (Figure 7C), indicating that the association between AKAPs and PKA_RII_ protects both proteins from degradation.

It is well established that intracellular pH is elevated during sperm capacitation [43]. Bicarbonate can alkalize the intracellular cytosol and we showed that intracellular alkalization, accelerates AKAP3 degradation rate (Figures 4A, 8c). Thus, bicarbonate has a dual effect on sperm, it can cause intracellular alkalization and can enhance cAMP by activation of soluble-adenylyl cyclase; both these effects are important for AKAP3 degradation. Changes in pH alter electrostatic interactions between charged amino acids and therefore we assume that alkalization may weakness PKA/ AKAP3 interaction resulting in AKAP3 degradation. Interestingly, intracellular alkalization, which enhances AKAP3 degradation, did not affect PKA_RII_ levels, and Ht31 caused relatively small degradation of RII compared to its effect on AKAP3 degradation (Figure 7C). Treatment with Ht31 or NH_4_Cl did not affect AKAP95 levels (Figure 7B) suggesting that AKAP3 is highly excitable to degradation compared to other AKAPs or to PKA_RII_. In order to explain the differential effect of intracellular alkalization versus treatment with Ht31 on RII degradation, we assumed that intracellular alkalization specifically affects AKAP3 degradation, causing a relatively small increase in unbound RII, whereas Ht31 induces the release of RII from all AKAPs, resulting in high levels of unbound RII, which is highly sensitive to degradation. The mechanism of AKAP3 degradation differs from that of PKCα and PP1γ2 degradation described by us elsewhere [38]. In both cases, cAMP enhances degradation, however, the PKA inhibitor H89 enhances AKAP3 degradation but inhibits PKCα or PP1γ2 degradation [38]. Since only partial degradation of sperm PKCα or PP1γ2 [38] occurs during capacitation, it is possible that only small proportion of these enzymes are associated with AKAP3 and their degradation depends on PKA activity.

AKAP3 binds a family of proteins sharing a conserved RII docking domain (R2D2) besides PKA_RII_, including ROPN1, ASP, SP17 and CABYR [20,44,45]. Binding of these proteins to AKAP3 is increased by AKAP phosphorylation, similarly to the increase in PKA_RII_ binding [20,28]. Selective binding of AKAP3 to phosphdiesterase-4A (PDE4A), which decomposes cAMP, has also been demonstrated in bovine sperm [46]. Thus, AKAP3 up-regulates PKA activity by targeting it to its site of activity, and down-regulates it by localization of PDE4A to this site. We assume that AKAP3 degradation would affect the activity of these proteins, while the physiological role of this degradation is still not clear. RhoA-interacting proteins are activated by phosphorylated-AKAP3 [20], and RhoA [47] as well as PKA [9] mediate actin polymerization in sperm capacitation. Thus, it is possible that AKAP3 is involved in an actin polymerization process that occurs during sperm capacitation.

Protein scaffold complexes are a key mechanism by which a common signaling pathway can serve many different functions. Sequestering a signaling enzyme to a specific subcellular environment not only ensures that the enzyme is near its relevant targets, but also segregates this activity to prevent indiscriminate phosphorylation of other substrates. Because AKAP3 is present mainly at the tail, it may be degraded towards the end of capacitation in order to direct PKA to other AKAPs that are localized in other areas of the sperm cell. PKA is involved in the AR, so AKAP3 degradation might cause a shift of PKA from the tail to the head in order to promote the AR.

In summary, we found that two factors affect AKAP3 degradation during sperm capacitation: dissociation between PKA and AKAP3 and intracellular alkalization. The results in this study support a physiological role of AKAP3 degradation in the capacitation process.
